# Silent voices: institutional disrespect and abuse during delivery among women of Varanasi district, northern India

**DOI:** 10.1186/s12884-018-1970-3

**Published:** 2018-08-20

**Authors:** Shreeporna Bhattacharya, T. K. Sundari Ravindran

**Affiliations:** 1 Jhpiego, Raipur, India; 2 Achutha Menon Centre For Health Science Studies, Sree Chitra Tirunal Institute for Medical Sciences and Technology, Thiruvananthapuram, Kerala 695011 India

**Keywords:** Abuse, Pregnant women, Institutional delivery, India, Health providers, Obstetric violence, Cross-sectional

## Abstract

**Background:**

A considerable amount of qualitative evidence reporting abusive treatment of women during delivery by health providers is available. However, there is a dearth of information regarding the actual prevalence and nature of such abuse, which this study aimed to explore.

**Methods:**

We conducted a community based cross-sectional study using a contextually adapted version of the Staha (meaning ‘respect’ in Swahili) project questionnaire among 410 rural women who delivered between June, 2014 to August 2015 at any health facility of Varanasi district, northern India. We selected the women through multi-stage cluster random sampling from two rural blocks of Varanasi, which recorded the highest number of institutional deliveries in 2014–15.

**Results:**

The frequency of any abusive behavior (excluding inappropriate demands of money due to its high prevalence-90.5%) was 28.8%. The reported abuses were non-dignified care including verbal abuse and derogatory insults related to the woman’s sexual behavior (19.3%); physical abuse (13.4%); neglect or abandonment (8.5%); non-confidential care (5.6%); and feeling humiliation due to lack of cleanliness bordering on filth (4.9%). Women were abused during labor or delivery irrespective of their socio-demographic background. Bivariate analysis using Chi-square tests showed statistically significant associations between abuse and provider type, facility type, and presence of complications during delivery. Binary logistic regression indicated that the odds of being abused was four times higher in those women who experienced complications during delivery. Though statistically insignificant, and contrary to expectations, women also seemed to be abused in private institutions; but with a lower frequency and of lesser severity.

**Conclusions:**

The prevalence of disrespect and abuse during labor or delivery was high among women irrespective of their socio-demographic background or delivery conditions in government as well as private health facilities. If the problem of disrespect and abuse is not addressed, it can be assumed that such harsh practices might promote home deliveries, which despite being more unsafe provide an empathetic environment in lieu of safe facility-based birthing options.

## Background

Ample literature exist which suggest that institutional deliveries and skilled care utilization can reduce maternal mortality [1–4]. With increasing rates of institutional delivery, the focus of policy attention is now shifting towards improving quality of care [5, 6]. One area of concern related to poor quality of care is the disrespect and abuse experienced by women in health care facilities during labour and delivery [7–10]. Such abusive practices represent a major violation of human rights. Realizing the negative impacts of disrespect and abuse of women in achieving skilled birth care, the WHO released a statement in 2014 stating ‘every woman has the right to the highest attainable standard of health, which includes the right to dignified, respectful health care’ throughout pregnancy and childbirth [10, 11]. Disrespectful and abusive treatment during childbirth not only violates the rights of women to a respectful care, but also threatens her right to life, health, bodily integrity, and freedom from discrimination [11]. Based on a comprehensive review of literature, Bowser and Hill conducted a landscape analysis identifying seven categories of disrespect and abuse: physical abuse, non-consented care, non-confidential care, non-dignified care, discrimination based on specific patient attributes, abandonment of care, and detention in facilities due to failure to pay [12]. These categories are often overlapping as a continuum and are not mutually exclusive. While there are clear evidence suggesting that abusive treatment of women has adverse impact on patient care and health outcomes [13–15], there have been limited studies assessing the nature and extent of disrespectful and abusive treatment of women during delivery in health care settings, especially in middle-income countries like India. A facility-based study conducted in the same geography as ours, showed under-reporting of disrespectful and abusive behavior by women who delivered at a public health facility. While 9.1% of women self-reported mistreatment during delivery, directly observed data was 22.4% [16].

We did our study in Varanasi – a district from the northern state of Uttar Pradesh in India. This state has one of the highest maternal mortality ratios (201 maternal deaths per 100,000 live births) [17] and institutional delivery rates (68%) in India [18]. The objectives of the study were to assess the frequency and nature of disrespect and abuse experienced in health care facilities by women, and explore possible associations, if any during labour and delivery. The intention is to draw policy attention to improve the quality of care provided to women who seek institutional delivery by incorporating the concept of respectful maternity care within the health system.

## Methods

### Study design, setting and sampling

We conducted a cross-sectional community based study in rural Varanasi district, northern India that has a population of more than 300,000. We estimated a sample size of 410 women assuming a prevalence of 20% [19], confidence level 95%, power 80%, design effect 1.5 and a non-response rate of 10%. We used multi-stage cluster sampling to choose two such *blocks* (which are the administrative units in a district) out of the eight which recorded the highest number of institutional deliveries in 2014 [20]. From each block, we randomly selected 20 women from 10 *panchayats* that are clusters of villages. From the 11th *panchayat*, we selected five women to complete the sample size. We included women from all age groups who were a) residing in the area for a minimum of 6 mos, b) had delivered at any government or a private health facility between June 2014 to August 2015 and c) were willing to participate in the study. The exclusion criterion was women with stillbirths or neonatal deaths.

### Study tool and data collection

The primary author collected the data between June–August 2015 using a survey tool in the vernacular language (Hindi), adapted from the tool used in the *Staha (*meaning ‘respect’ in Swahili) project after piloting with 30 women^a^. The pilot helped in removing certain questions on sexual abuse from the *Staha* tool (that got poor responses from women), and to see the feasibility of asking sensitive questions on disrespect and abuse to a vulnerable group of rural Indian women. We also explored time required to spend in each household and ways to contact and initiate conversations with such pregnant women who were abused. The tool was a structured interview schedule with closed and open-ended questions about demographic characteristics, socio-economic status, health history, recent health-care utilization, delivery characteristics, perceived health care quality and satisfaction, experiences of disrespect and abuse during delivery and future health care utilization.

We measured disrespect and abuse by asking women whether they experienced specific events during their labour and delivery. The disrespect and abuse items in the study tool were:

- **Physical abuse:** slapping, pinching, beating or tying the women; invasive procedures done without any pain relief (episiotomies and caesarean sections); repetitive use of excessive force during vaginal examinations/labour/delivery)
- **Non-dignified care:** shouting/ scolding; threatening to withhold treatment; threatening or negative comments (in the form of rude remarks about the woman’s sexual behavior that led to pregnancy)
- **Neglect and abandonment:** ignoring or abandoning the women when in need and/or delivering alone
- **Non-confidential care:** lack of physical privacy (that is, body seen by non-health personnel); disclosing private health information to others without consent and doing procedures like tubal ligation, caesarean sections and hysterectomy without consent
- **Non-consented care:** doing tubal ligation; caesarean sections and hysterectomy without consent of the woman or her family members
- **Inappropriate demands for money:** demands for bribe for better care and detaining mother or the baby or both in facilities due to failure to pay
- **Other types of disrespect and abuse:** an open ended category was kept to capture any other kind of disrespect and abuse that women experienced during their delivery

### Statistical analysis

We did descriptive analysis of all the socio-demographic characteristics and delivery experiences of the pregnant women. We created a composite variable with two categories - poor and non-poor to measure the economic status using three variables namely type of house, owner of Below Poverty Level card (which is a type of food ration card issued by the state government to all those families living below the poverty line) and difficulty in meeting monthly household expenditures. We did binary coding for each sub-category of abuse. Although some of the items on disrespect and abuse overlapped in meaning, we included them nonetheless as multiple responses may represent a single abuse incident. Responses to each sub-category listed within the major categories of abuse were given a binary code (abused = 1 and non-abused = 0). When collated, we gave codes to major types of abuse (physical abuse, non-dignified care, non-confidential care, non-consented care, neglect and abandonment, inappropriate demands of money and other types) in to ‘abused’ if any of the sub-categories were recorded as ‘abused’. We calculated ‘any abuse’ (representing any type of abuse experienced by the woman) as a composite variable including all the major types of abuse by excluding ‘inappropriate demands of money’ from the group. We measured overall quality of care and satisfaction with delivery as ‘excellent/very good, good and fair/ poor.

We first did bivariate analysis to exclude statistically insignificant predictors through Chi-square tests followed by a binary logistic regression to explore associations between predictor variables and abuse (alpha value-5%). We performed statistical analyses using SPSS version 22 (IBM). We further probed those women who reported disrespect and abuse through various open-ended questions. We transcribed, read and re-read the testimonies in the open-ended questions to do detailed thematic analyses for deducing other forms of abuse not captured through quantitative questions.

## Results

### Socio-demographic and delivery characteristics

Among 415 women who were available and met the inclusion criteria, five did not consent to be a part of the study and were classified as ‘non-responders’ making the final sample of 410 respondents (response rate of 98.78%).

Table 1 shows the socio-demographic and the delivery characteristics of the women. The mean age of the women was 24.7 years (Standard Deviation (SD) = 3.2). The majority were Hindu (94.9%) and more than 80% were from low-income households. More than 40% were from socially marginalized *dalit* castes and most of them were homemakers (*N* = 351, 85.6%) despite being educated (*N* = 333, 81.2%). On average, a woman had 2.32 (SD-1.34) deliveries in her lifetime (range 1–9).

**Table 1:** Socio-demographic profile and delivery details of women in the study sample 2015

Variables	*N* = 410 (%)
Age - Mean (in years) ± SD	24.7 (3.18)
Educational status	
No education	77 (18.8)
< = Secondary (till class X or less)	197 (48.0)
> =Higher secondary and above (class XII or above)	136 (33.2)
Occupational status	
Remunerated Work	54 (13.2)
Non-remunerated work	356 (86.8)
Place of delivery	
a) Government facilities	
Primary Health Center	86 (21.0)
Community Health Center	53 (12.9)
District Hospital/ Tertiary Hospital	9 (2.2)
Health Sub-Centre	135 (32.9)
Other government facilities	19 (4.6)
b) Private facilities	108 (26.3)
Previous use of facility before the current delivery	
Yes	215 (52.4)
No	195 (47.6)
Companion^3^ present during delivery	
Yes	251 (61.2)
No	159 (38.8)
Main provider conducting delivery	
Doctor	135 (32.9)
Nurse	231 (56.3)
Informal worker^4^	40 (9.8)
None	4 (1)
Type of delivery	
Normal/ episiotomies	354 (86.3)
C-section/ Vacuum extraction/ forceps delivery	56 (13.7)
Complications post-delivery	
Yes	65 (15.8)
No	345 (84.1)
Overall quality of care during delivery	
Very good	45 (10.9)
Good	242 (59.0)
Fair/ Poor	123 (30.0)
Choice of facility for next delivery	
Same facility	183 (88.8)
Another facility	23 (11.2)

^3^relatives/ neighbours/ friends

^4^ASHA/ traditional attendants/ other non-skilled worker

Nearly three out of every four women (*N* = 302, 73.7%) had delivered at government facilities and even among them, 135 (44.7%) delivered at local health facilities called ‘Health Sub-Centres’. More than half of the women responded going to their respective facilities because of their vicinity (*N* = 262, 63.9%), low cost (*N* = 198, 48.3%) and familiarity (*N* = 134, 32.7%). In terms of familiarity with the health facility, around half (*N* = 215, 52.4%) of the women had gone to the facility earlier and for their previous deliveries (*N* = 138, 64.2%). Almost everybody was escorted to the facility, but only 251 (61.4%) had companions during delivery.

More than half (56.3%) of the health providers attending delivery were female nurses. Deliveries were predominantly normal (*N* = 354, 86.3%) and hence, most of the women (*N* = 330, 80.5%) did not stay for more than 1 day at the health-facility after delivery. Despite around 65 (15.9%) women reporting complications post-delivery, most reported the overall quality of services to be ‘good’ (*N* = 242, 59.0%).

### Disrespect and abuse

Table 2 depicts disrespect and abuse experienced by women through health providers during their delivery. We excluded inappropriate demands of money (90.5%)’ and ‘non-consented care (0%)’ while calculating ‘any abuse’ as being outliers, they would have skewed the association analysis. Excluding these forms of abuse, proportions of women who experienced any disrespect or abuse was 28.8%. Around 9% of the respondents who reported ‘any abuse’ had experienced more than one type of abuse. Nearly 13.4% pregnant women who underwent physical abuse reported being slapped or pinched (2.7%); put to excessive force during examination or delivery (12%) and have undergone procedures without any pain relief (1.5%). Around 19.3% of the respondents reported receiving non-dignified care in forms of shouting or scolding (17.3%); threats to withholding treatment if could not pay or did not have supplies (4.1%) and being threatened or humiliated through rude sexual remarks in public (6.3%). Almost 8.5% of the respondents were ignored when asked for help and two among them delivered without any assistance of a health care provider. Further, 5.6% of the women underwent vaginal examinations or deliveries in presence of strangers without any curtains or physical privacy. Around 13.2% of respondents were detained in the facilities and were not given their babies if the desired amount demanded by the staff was paid. Additionally, 20 (4.9%) women considered prevailing unhygienic conditions and lack of basic amenities also as a type of abuse which was not mentioned in the tool. This category was explored through further probing during the qualitative interviews, and was mentioned as ‘other abuse’ during analysis (see Table 3).

**Table 2:** Disrespect and abuse of women in the study sample, 2015

Variables	*N* = 118 (%)	
Frequency of abuse	*Any abuse* ^a^	*Any abuse* ^b^
Never abused	30 (7.3)	292 (71.2)
Experienced only 1 type of abuse	266 (64.9)	57 (13.9)
Experienced 2 types of abuse	56 (13.7)	39 (9.5)
Experienced 3 types of abuse	37 (9.0)	13 (3.2)
Experienced = > 4 types of abuse	21 (5.12%)	9 (2.2%)
Any disrespect and abuse	380 (92.7)	118 (28.8)
Specific experiences of disrespect and abuse excluding inappropriate demands of money (overlapping categories)		
Physical abuse		
Overall	55 (13.4)	
Physical abuse (slapping/ pinching etc.)	11 (2.7)	
Use of excessive force during delivery	49 (12)	
Delivery without any pain relief	6 (1.5)	
Non-dignified care		
Overall	79 (19.3)	
Shouting/scolding	71 (17.3)	
Threat of withholding treatment	17 (4.1)	
Threatening or negative comments	26 (6.3)	
Non-confidential care		
Overall	23 (5.6)	
Disclosing private health information to others	0 (0)	
Delivery without any physical barriers	23 (5.6)	
Neglect		
Overall	35 8.5)	
Ignored when needed help	35 (8.5)	
Delivery without attendant	2 (0.5)	
Inappropriate demands for money		
Overall	371 (90.5)	
Detention in facility for failure to pay	54 (13.2)	
Request for bribe	371 (90.5)	
Other forms of abuse	20 (4.9)	

^a^abuse including inappropriate demands of money; ^b^abuse excluding inappropriate demands of money

**Table 3:** Case series of different types of disrespect and abuse

*Physical abuse* *‘They were inserting their fingers inside me in every 10 min (….) it was so painful. Then they would beat me up on the thighs. They did that several times (….) during delivery I would cry (….) I do not how many they were, but almost everybody had pressed me down to the bed, I could not move (….) The moment I drew my breath, everybody became so mad at me. They started beating me more, pushing on my abdomen. It was so painful that it got torn down there. While giving the stitches, they did not give me anything for pain. It was my first delivery and now I am really scared for my second child’ -* *Primi mother from a lower caste* *Non-dignified care* ***‘*** *When you were sleeping with your husband, then you did not scream, and now you are? (….) If you do not want babies but want to have fun then do ligation’ -* *Mother of three from a lower caste* *Neglect and abandonment* *‘They gave me a bed but nobody came to look after me (…) they were so busy with some other woman that my mother took the curtains from the windows as we did not have any plastic sheet with us (…) she (nurse) started pushing on my abdomen (…) by the grace of God, I delivered and it was a daughter’ -* *Mother of three from a lower caste* *Non-confidential care* *‘I was lying on the bed naked drenched in my own blood after delivery for around half an hour in the presence of everybody (…) I somehow found my clothes and covered myself’ -* *Mother of two from a lower caste* *Inappropriate demands of payment* *‘The nurse told everybody has to pay, and then only you will get the money from the government. Also, all the medicines were bought with my money, so that has to be covered as well. Give 2000 rupees, else I will not give your baby’-* *Mother of two from a higher caste* *Other types of abuse (lack of cleanliness)* *‘It was like a dump. I could see blood and stuff of other women on the bed sheets. There were plastic bags, used sanitary napkins, urine, blood, vomit, food; everything on the floor (…) the toilets were overflowing with faeces with no water facility inside (…) finally I had to go to the fields’ -* *Mother of five from a lower caste*	

## Factors associated with disrespect and abuse

We performed Chi-Square and Fischer’s Exact tests to determine bivariate associations between various predictor variables and abuse (Table 4). Despite being statistically insignificant, one interesting finding was that women were disrespected and abused in government and private health facilities equally (30% and 25% respectively). Type of provider, facility type and presence of complications were statistically significant in bivariate analysis.

**Table 4:** Bivariate analysis between selected predictor variables and disrespect and abuse

Predictor variable	% of pregnant women abused	*p*-value
Age		
< 25 years	29.1	0.87
= > 25 years	28.4	
Education		
= <Secondary education (till class X)	27.4	0.69
> Secondary education (more than class X)	29.4	
Occupation		
Non-remunerated work	27.5	0.15
Remunerated work	37.0	
Caste		
General	29.6	0.15
OBC^a^	27.6	
SC/ST^b^	29.2	
Economic status		
Non-poor	29.4	0.96
Poor	29.1	
Total number of children		
One child	29.9	0.94
2–3 children	28.4	
= > 4 children	27.7	
Type of facility		
Government	30.1	0.31
Private	25.0	
Nature of government facility		
PHC^c^ and HSC^d^	24.9	0.00*
CHC^e^	47.2	
DH/ TH^f^	55.6	
Referred from first facility approached		
Yes	36.4	0.18
No	27.6	
Respondent was escorted for current delivery to the facility		
Yes	29.1	0.20
No	0	
Companion present during delivery		
Yes	30.3	0.40
No	26.4	
Type of provider conducting delivery		
Doctor	36.3	0.04^*^
Nurse/ ANM^g^	23.8	
ASHA^h^/ *Dais*	27.5	
Presence of complications		
Yes	44.6	0.00^*^
No	25.8	

*p-value in Chi-square test < 0.05; ^a^-Other Backward Class; ^b^-Scheduled Caste/ Scheduled Tribe; ^c^-Primary health Centre; ^d^-Health Sub-centre; ^e^CHC-Community Health Centre; ^f^-District Hospital/ Tertiary Hospital; ^g^-Auxilliary-Nurse Midwife; ^h^ASHA-Accredited Social Health Activist;

We ran a binary logistic regression with the statistically significant predictor variables derived from bivariate analysis to explore further associations with abuse (Table 5). Regression analysis showed that when adjusted for the provider and facility type, the odds of being abused was four times higher in those pregnant women who experienced abuse. The lack of significant statistical association of disrespect and abuse with most of the predictor variables indicates that abuse is a widespread phenomenon and occurs without any obvious correlation.

**Table 5:** Binary logistic regression analysis between selected predictor variables and disrespect and abuse

Predictor variable	Adjusted Odds Ratio (95% CI)
Presence of complications	
Yes	4.18 (1.78–9.83)
No	Ref
Type of provider	
Doctors	2.56 (0.89–7.36)
Nurses	0.75 (0.32–1.76)
ASHA^h^/ *Dais*	Ref
Type of facility	
PHC^c^ and HSC^d^	Ref
CHC^e^	1.00 (0.19–5.30)
DH/ TH^f^	2.52 (0.45–14.07)

^c^Primary Health Centers

^d^Sub-Health Centers (Note: HSC should be SHC)

^e^Community Health Centers

^f^District Hospital/ Tertiary Hospital

^h^Accredited Social Health Activist

## Discussion

The aim of this study was to find the nature and extent of disrespect and abuse experienced by women by the providers during institutional deliveries. Our study is among the very few stand-alone studies from India that have systematically documented abusive practices on women in health-care facilities indicating a troublesome picture of the quality of care women received during labour and/ or delivery.

The prevalence of abuse excluding inappropriate demands of money was 28.8%. In our study, non-dignified care and physical abuse were the most common types of abuse. A similar study was conducted in Gujarat, India among women from urban slums who had delivered at the government facilities reporting non-consented care (57.3%) and verbal abuse (55%) as the most common types of abuse [21]. A possible explanation for the contrasting results can be the use of different definitions for each kind of abuse and because the sample was restricted only to the government facilities. In another facility-based study, observation data showed that 22.4% women were mistreated during delivery. Primi, women with complications, and attended by unskilled providers were abused more [16].

Bivariate analysis showed that doctors were more abusive. In addition, those who experienced complications during delivery were abused more. Though we did not explore this association, it can be assumed that doctors were more involved in treating complicated cases. A high patient load at the facilities, particularly a higher-level facility where most of the complicated cases were referred could have possibly contributed in amplifying the abusive behavior of the providers. In our study, nearly four out of nine pregnant women who were abused delivered at a district hospital or tertiary hospital. Other studies have shown that higher level public facilities in India are inadequate in numbers, ill-equipped and short of trained providers as per the population coverage norms [22].

From the mother’s perspective, having a safe delivery is the biggest priority and she could not have reported any abuse if her child was delivered safely. That could have been the reason that a statistically significant association was present between quality of care and presence of complications with nearly half of the women experiencing complications reported they received ‘fair/ good’ quality of care. Our regression analyses results indicated that women who had complications during delivery were abused more than their counterparts were. This is an important finding as maternal stress of any kind stalls the labor process, thereby increasing the chances of complications [23]. So such kinds of abusive provider behavior that can cause complications should be addressed to improve maternal health outcomes.

We found studies with similar results as ours. For example, in South Africa, women were frequently physically abused [24]. In Tanzania, women experienced frequent and unnecessary vaginal examinations to the extent that the vulva got swollen, preventing normal progress of labour. At the same time, frequent vaginal examinations by different people presented a serious risk of infection [25–27]. In our study, women did not complain about the risk of infections but talked of feeling humiliated as a result of being checked repeatedly by multiple providers in the presence of strangers. Similar findings corroborating to our study were found in Kenya where invasive procedures like episiotomies / suturing of episiotomies without any pain relief were conducted and derogatory remarks on the sexual life of woman were used [28, 29]. It is not clear whether this was a consequence due to the non-availability of supplies or an intentional act on part of the providers.

An equally interesting finding, though statistically insignificant was the high prevalence (25%) of abuse in private health facilities compared to the government facilities (30.1%). This shows that abusive provider behavior has become a norm and is not restricted to only government facilities where the providers are over-worked and have to work with limited resources.

### Strengths and limitations of the study

This study had a number of strengths. It was a unique community based study in India quantitatively exploring disrespect and abuse of women by providers in an institutional setting. We used a validated instrument with many open-ended questions to cross-validate the quantitative questions and explore other forms of abuse. The random selection of clusters and women rendered the sampling bias free and being a single investigator study, there were no inter-observer biases. The chances of recall bias were also limited as it we presume that an extreme event like abuse during delivery could not be forgotten easily. Being self-reported, this study might not have exhaustively captured all cases of abuse. However, given that the negative consequences of abuse for women are mediated by their own view of what is abusive, self-reported data was thought to be the most appropriate measure. Since, different kinds of abuse can occur simultaneously, many categories of abuse were overlapping. We could have explored better associations if we had a larger sample, which was not possible due to budget and time constraints. Additionally, this study was specific to the demographic and cultural context of Varanasi, and hence, could not be generalized to all situations.

## Conclusions

This study indicates a significant frequency of disrespect and abuse of women during institutional childbirth. There were no apparent correlations between socio-demographic or delivery characteristics except for the presence of complications during delivery. Regardless of the circumstances and causes, abuse of women during delivery must be viewed as a violation of human rights jeopardizing their health and preventing them from enjoying a respectful and dignified childbirth. Until now, a vertical approach was followed for all the maternal health policies in India. However, to support quality improvements and increased access to service, a rights-based approach should be followed; promoting the active participation of women in all aspects of their own care; where the women are treated not as subordinates but as equals. Additionally, there should be proper accountability measures for directly addressing the inequities in power between the providers and women starting with setting a national rights-based surveillance system on maternal health based on culturally competent and women friendly guidelines. More mixed-method studies with larger sample size studies exploring the health system limitations, provider behavior and the experiences of women are needed by using the tool used in this study or developing a new or modified tool to come up with a single and unified definition of abuse.

## Abbreviations

ANM: Auxiliary nurse midwife
ASHA: Accredited social health activist
CHC: Community health centre
DH/ TH: District hospital/ tertiary hospital
FE: Forceps extraction
HSC: Health sub-centre
LMICs: Lower and middle income countries
OBC: Other backward classes
PHC: Primary health centre
SC/ST: Scheduled caste/ scheduled tribe
SPSS: Statistical package for the social sciences
VE: Vaginal examination
WHO: World Health Organization
